# Editorial: Effects and mechanisms of probiotics, prebiotics, synbiotics and postbiotics on intestinal health and disease

**DOI:** 10.3389/fcimb.2024.1430312

**Published:** 2024-06-24

**Authors:** Guangqiang Wang, Tongren Ding, Lianzhong Ai

**Affiliations:** Shanghai Engineering Research Center of Food Microbiology, School of Health Science and Engineering, University of Shanghai for Science and Technology, Shanghai, China

**Keywords:** probiotics, prebiotics, synbiotics, postbiotics, mechanisms

Probiotics, a collective term for microorganisms with health benefits, are known for their ability to regulate the gut microbiota and boost the immune system. Substances closely related to probiotics, such as prebiotics and postbiotics, also play a significant role in maintaining intestinal health and treating certain diseases. Given the substantial impact of these substances on the gut microbiota and overall human health, we have compiled a selection of popular research articles from the journal “Frontiers in Cellular & Infection Microbiology”, focusing on the theme “Effects and Mechanisms of Probiotics, Prebiotics, Synbiotics, and Postbiotics on Intestinal Health and Disease.” By analyzing these articles, we aim to elucidate the roles and effects of these substances in promoting intestinal health and treating specific illnesses, with the goal of providing scientific insights into their mechanisms and applications.

## The application of probiotics in disease treatment

Probiotics have been considered to have significant potential in the treatment of certain diseases, such as alcoholic liver disease (ALD), but there is still widespread controversy. ALD can be alleviated through nutritional support and alcohol abstinence, treatment strategies remain relatively limited. Xiong et al. conducted a systematic review and meta-analysis to thoroughly evaluate the clinical efficacy of probiotics in treating ALD. The results indicated that probiotic formulations can significantly improve liver function indicators in patients with ALD, reducing serum levels of alanine aminotransferase (ALT) and aspartate aminotransferase (AST), which reflect liver inflammation and injury, and increasing the levels of serum albumin, which reflects liver synthetic function. The modulation of the gut microbiota by probiotics is likely key to their therapeutic effect. By increasing the number of beneficial bacteria and decreasing the number of harmful bacteria, probiotics help to maintain a balanced gut microbiota. This balance reduces the production of inflammatory mediators, thereby mitigating liver damage and promoting liver repair. However, current research has certain limitations, such as inconsistent intervention times, dosages, and small sample sizes. Future studies could more accurately assess the efficacy of probiotics in ALD treatment by expanding sample sizes and standardizing intervention times. Additionally, further research is needed to clarify the mechanisms of interaction between probiotics and the gut microbiota, as well as their impact on liver function and metabolic pathways.

## The potential of the new generation of probiotics in the treatment of psychiatric disorders

In recent years, some studies have identified a new generation of probiotics, such as *Akkermansia muciniphila*, which may have potential value in the treatment of psychiatric disorders. Psychiatric disorders, including depression and anxiety, are complex conditions influenced by a combination of genetic, lifestyle, and environmental factors. Traditional treatments, such as medication and psychotherapy, have shown less than ideal outcomes, facing challenges like significant individual differences, side effects, and relapse. Lei et al. reviewed the positive role of *A. muciniphila* in the treatment of psychiatric disorders. Studies suggest that this probiotic interacts with the brain through the microbiota-gut-brain axis, potentially influencing the signaling between the gut and the brain by producing metabolites such as short-chain fatty acids, thereby exerting a positive impact on mental health. These findings offer a new perspective and potential therapeutic mechanisms for the treatment of psychiatric disorders. However, the use of the new generation of probiotics in the treatment of psychiatric disorders still faces many challenges. Currently, data on their efficacy and safety are insufficient, and more large-scale, long-term, high-quality clinical studies are needed to verify their effectiveness. Moreover, there are differences in individual responses to probiotics, and future research should develop personalized treatment plans to improve therapeutic outcomes and reduce adverse reactions, making probiotic treatment more precise and effective. In addition, further research is needed on the mechanisms of action, efficacy, and safety of probiotics to provide a scientific basis for their use in clinical treatment, thereby offering new treatment options for patients with psychiatric disorders.

## The regulatory effect of synbiotics on hypertension

Synbiotics, a combination of probiotics and prebiotics, have shown greater potential in improving intestinal function and enhancing host health compared to probiotics alone. In the field of disease treatment, particularly in the non-pharmaceutical management of hypertension, the application of synbiotics is increasingly gaining the attention of researchers. Hypertension is a chronic condition primarily treated with medication, which often comes with certain side effects such as fatigue and gastrointestinal discomfort. Chen et al. conducted a comprehensive analysis and reviewed the alleviating effects of synbiotics on hypertension. The article indicates that synbiotics can exert their effects through various mechanisms, such as regulating the gut microbiota, improving vascular oxidative stress, reducing inflammatory responses, and promoting the production of short-chain fatty acids, which significantly lower blood pressure levels in hypertensive patients and thus promote vascular health. These studies highlight the potential of synbiotics in reducing blood pressure and provide new strategies for the management and treatment of hypertension. However, there are still some issues in the clinical application of synbiotics, such as individual differences, the ratio of synbiotic components, and long-term safety assessments. Future research needs to further explore the personalized application of synbiotics, their long-term effects, and mechanisms of action. In addition, to better apply synbiotics in disease treatment, more clinical studies are needed to verify their efficacy and safety, providing safe and effective treatment options for patients with hypertension.

## The antimicrobial activity and application of postbiotics

The improper use of antibiotics has led to an increase in bacterial resistance, greatly diminishing the therapeutic efficacy of antibiotics and disrupting the balance of the gut microbiota, which can lead to gastrointestinal dysfunction and other issues. Therefore, it is particularly important to seek new treatment methods to mitigate these effects. Penchuk et al. conducted research that found the bacterial lysates of *Lactobacillus rhamnosus* exhibited significant antibacterial activity against a variety of bacteria and yeasts, with a minimum inhibitory concentration (MIC) value significantly lower than that of the heat-inactivated control group. Notably, when this postbiotic was used in conjunction with blackcurrant extract, its antibacterial effect was even more pronounced. This discovery not only highlights the potential of postbiotics in antibacterial applications but also reveals the possible synergistic effects between postbiotics and plant extracts, providing a reference for reducing antibiotic resistance. In the future, further in-depth exploration is needed regarding the optimal dosage of postbiotics, the safety of long-term application, and how to develop personalized treatment plans. This will help to effectively supplement antibiotics with postbiotics, alleviating problems caused by the misuse of antibiotics, promoting intestinal health, and improving the quality of life for patients.

## Final considerations

With the deepening of related research, the potential of probiotics, synbiotics, and postbiotics in disease treatment is gradually being discovered. They have shown good effects in improving intestinal health, regulating microbial communities, and enhancing immunity, such as in the treatment of alcoholic liver disease, psychiatric disorders, and hypertension. In addition, the antimicrobial properties of postbiotics also provide new ideas for reducing antibiotic resistance. However, future research still needs to further verify their long-term safety and efficacy, and explore the optimal therapeutic dosages and personalized treatment plans.

## Author contributions

GW: Writing – original draft, Writing – review & editing. TD: Funding acquisition, Writing – original draft. LA: Funding acquisition, Writing – review & editing.

